# Mesocardia With Upward and Leftward Apex Secondary to a Large Hiatal Hernia: A Case Report

**DOI:** 10.7759/cureus.97821

**Published:** 2025-11-26

**Authors:** Suzan Iskandar, Abbas Rachid, Joe Hitti, Malek Mohammed, Hasan Kazma

**Affiliations:** 1 Cardiology, Lebanese University, Beirut, LBN; 2 Internal Medicine, Lebanese University, Beirut, LBN; 3 Department of Cardiology, Bahman University Hospital, Beirut, LBN

**Keywords:** cardiac axis deviation, cardiac displacement, cardiac positional anomaly, dextro-rotation, subtle dextrocardia

## Abstract

We report the case of an 82-year-old woman with a known history of hiatal hernia who presented with intermittent chest discomfort and exertional dyspnea. Electrocardiography revealed sinus rhythm with axis deviation and a prominent R wave in lead aVR, initially raising concern for limb lead reversal, conduction abnormality, or myocardial ischemia. Chest radiography demonstrated a retrocardiac air-fluid level, and computed tomography (CT) confirmed a large type III hiatal hernia displacing the heart centrally, with the apex directed upward and leftward. No congenital structural cardiac abnormalities were identified. The patient was managed conservatively. This case highlights how a large hiatal hernia can alter cardiac orientation and produce atypical electrocardiographic findings. It underscores the importance of correlating ECG abnormalities with imaging studies to avoid misdiagnosis and unnecessary interventions.

## Introduction

Hiatal hernias are common, and they usually cause digestive symptoms such as reflux or chest discomfort. However, when the hernia becomes large enough to enter the thoracic cavity, it can affect structures far beyond the esophagus and stomach. By occupying space in the chest, the herniated stomach can push the heart out of its normal position, lifting it upward, rotating it, or shifting it toward the right side. These mechanical changes can modify the direction of the heart's electrical forces, even though the myocardium itself is completely normal [1,2].

In the elderly, alterations in cardiac position may also arise from extracardiac causes, including large hiatal hernias, which can shift mediastinal structures and lead to atypical electrocardiographic findings [3,4].

We present the case of an elderly woman who presented with chest discomfort and abnormal electrocardiographic changes, in whom imaging revealed a large hiatal hernia pushing the heart upward toward the right shoulder.

## Case presentation

An 82-year-old woman with a history of hypertension, coronary artery disease, diabetes mellitus, and gastroesophageal reflux disease presented with several weeks of intermittent chest discomfort, exertional dyspnea on minimal activity, and postprandial fullness. She denied palpitations, presyncope, or syncope. On arrival, her vital signs were stable, and her physical examination was unremarkable.

**Figure 1:**
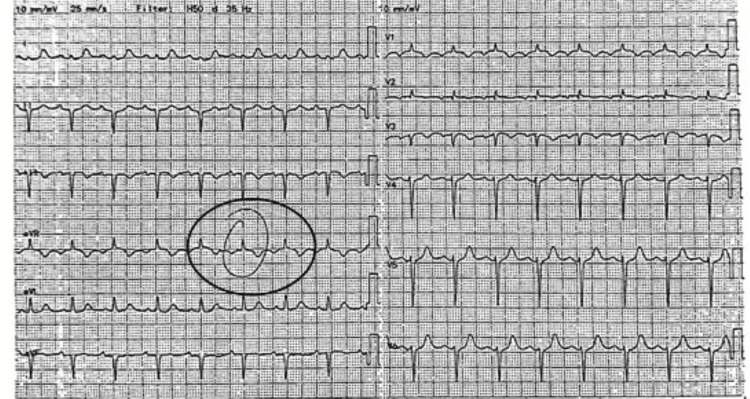
ECG demonstrating sinus rhythm with a prominent R wave in lead aVR (circle), low limb-lead voltages, and left axis deviation. ECG: electrocardiogram, aVR: augmented voltage right arm

Electrocardiography (ECG) revealed sinus rhythm with a tall R wave in lead augmented vector right (aVR), low limb-lead voltages, and left axis deviation (Figure 1). Initial considerations included lead misplacement or myocardial ischemia; however, serial ECGs showed no changes, and cardiac biomarkers were negative, prompting evaluation for possible anatomical displacement.

**Figure 2:**
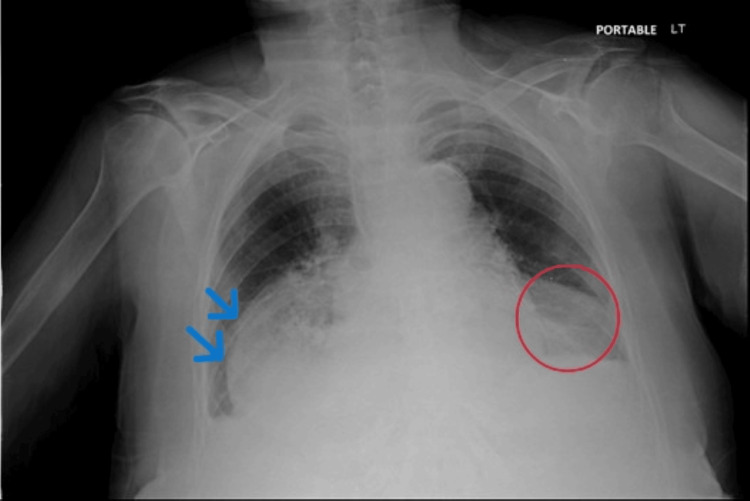
Chest X-ray demonstrated a large retrocardiac air-fluid level consistent with a hiatal hernia (blue arrows), along with a mildly right-shifted cardiac silhouette (red circle).

Chest radiography demonstrated a retrocardiac air-fluid level consistent with a large hiatal hernia, along with a mildly right-shifted cardiac silhouette (Figure 2).

**Figure 3:**
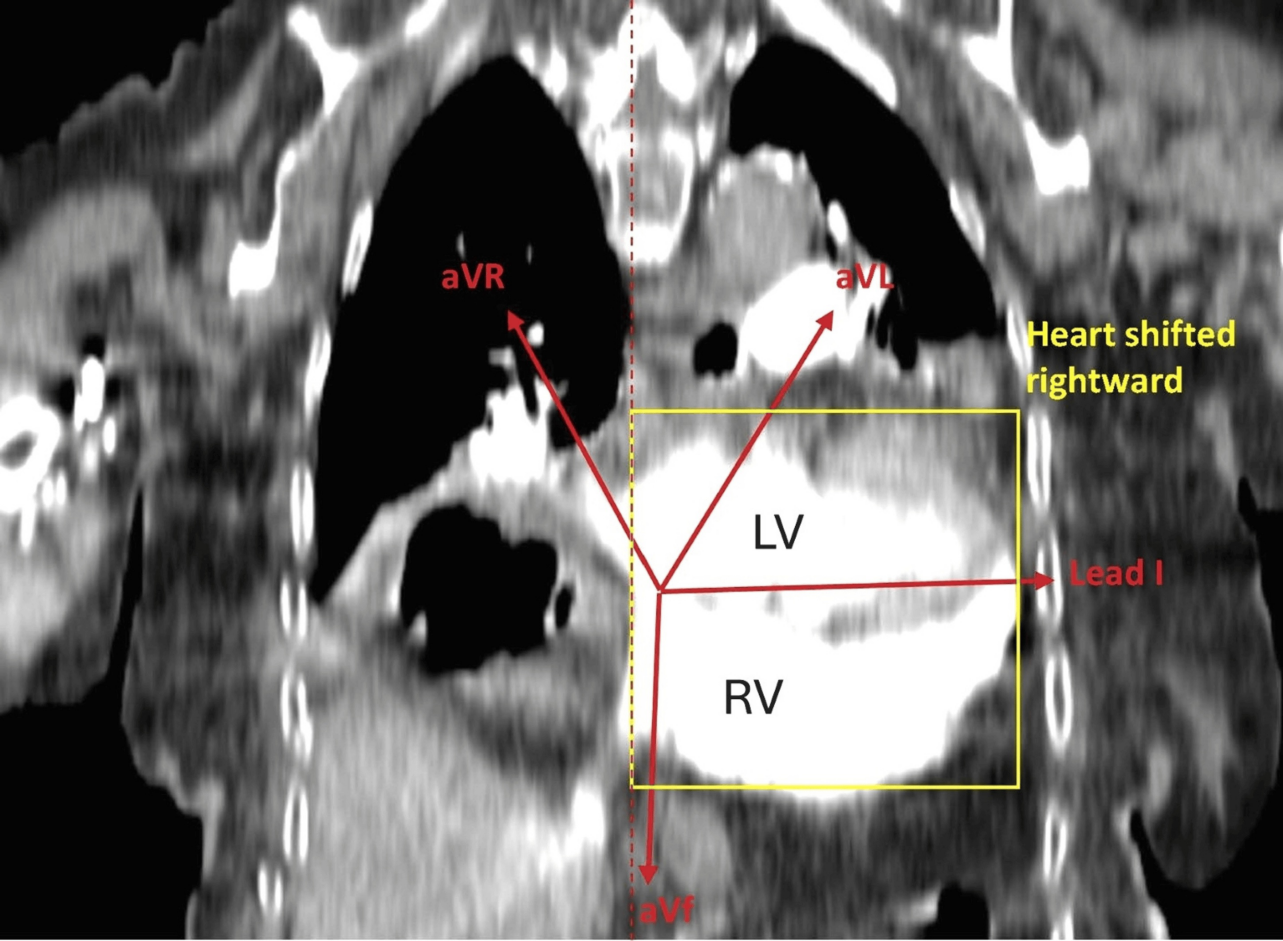
CT Coronal view of cardiac anatomical orientation with ECG lead vector projections demonstrating upward and rightward apex with a positive aVR vector projection. LV: left ventricle, RV: right ventricle, ECG: electrocardiogram, aVF: augmented voltage foot, aVL: augmented voltage left arm, aVR: augmented voltage right arm

**Figure 4:**
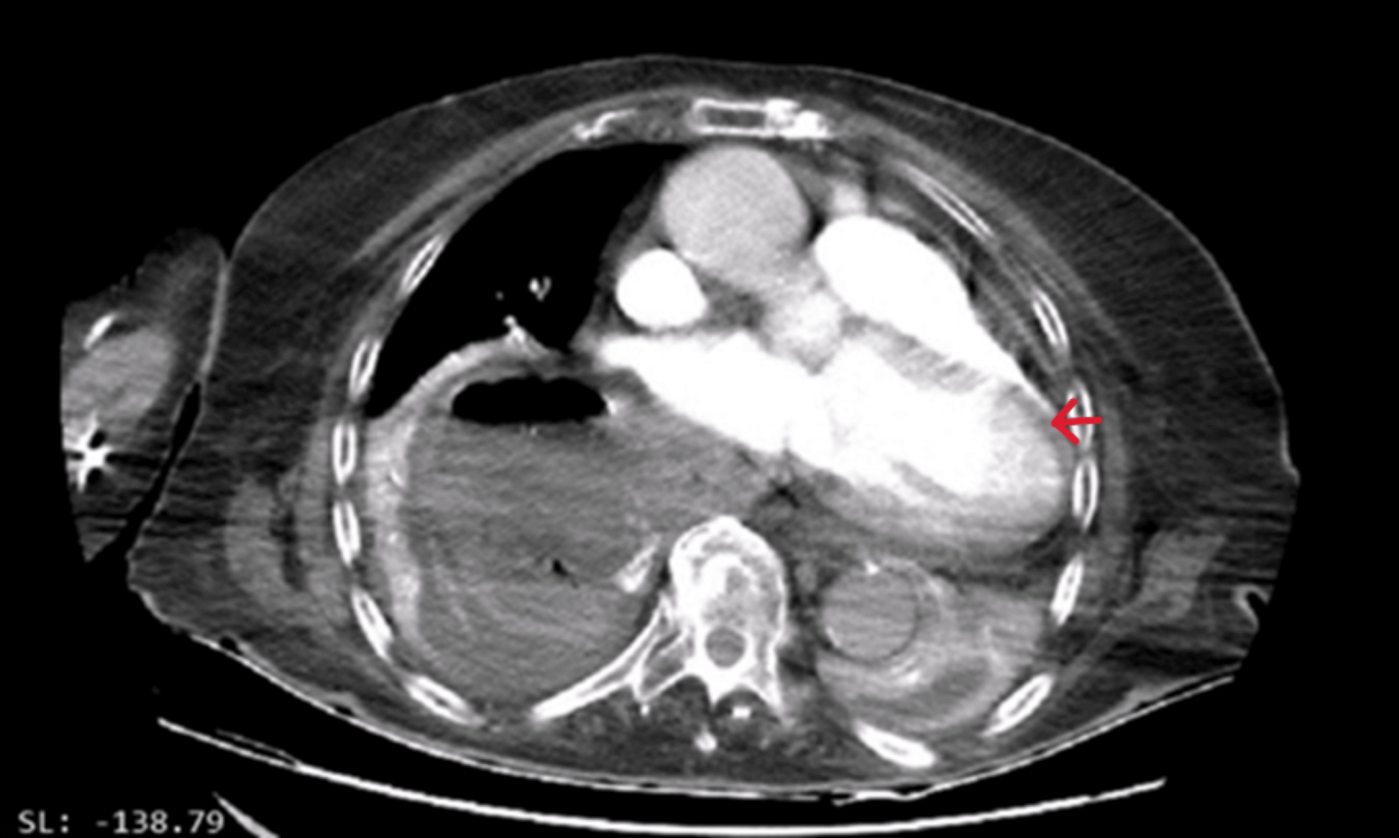
Axial view of Angio CT scan demonstrating a cardiac apex positioned (red arrow) posteriorly and slightly to the right.

Contrast-enhanced computed tomography (CT) confirmed a large type III hiatal hernia displacing the heart superiorly and rightward. Coronal and axial views showed the cardiac apex directed posteriorly and slightly rightward (Figures 3, 4).

The cardiac chambers were normal, with no evidence of situs inversus or structural congenital abnormalities. Both atria and ventricles were appropriately located, and the great vessels, including the aorta and pulmonary artery, arise from their expected positions and followed a normal course. The long axis of the heart appeared more vertical and posterior than usual, deviating from the typical leftward and anterior orientation. The sternum, thoracic spine, and diaphragm were intact and normally aligned.

**Figure 5:**
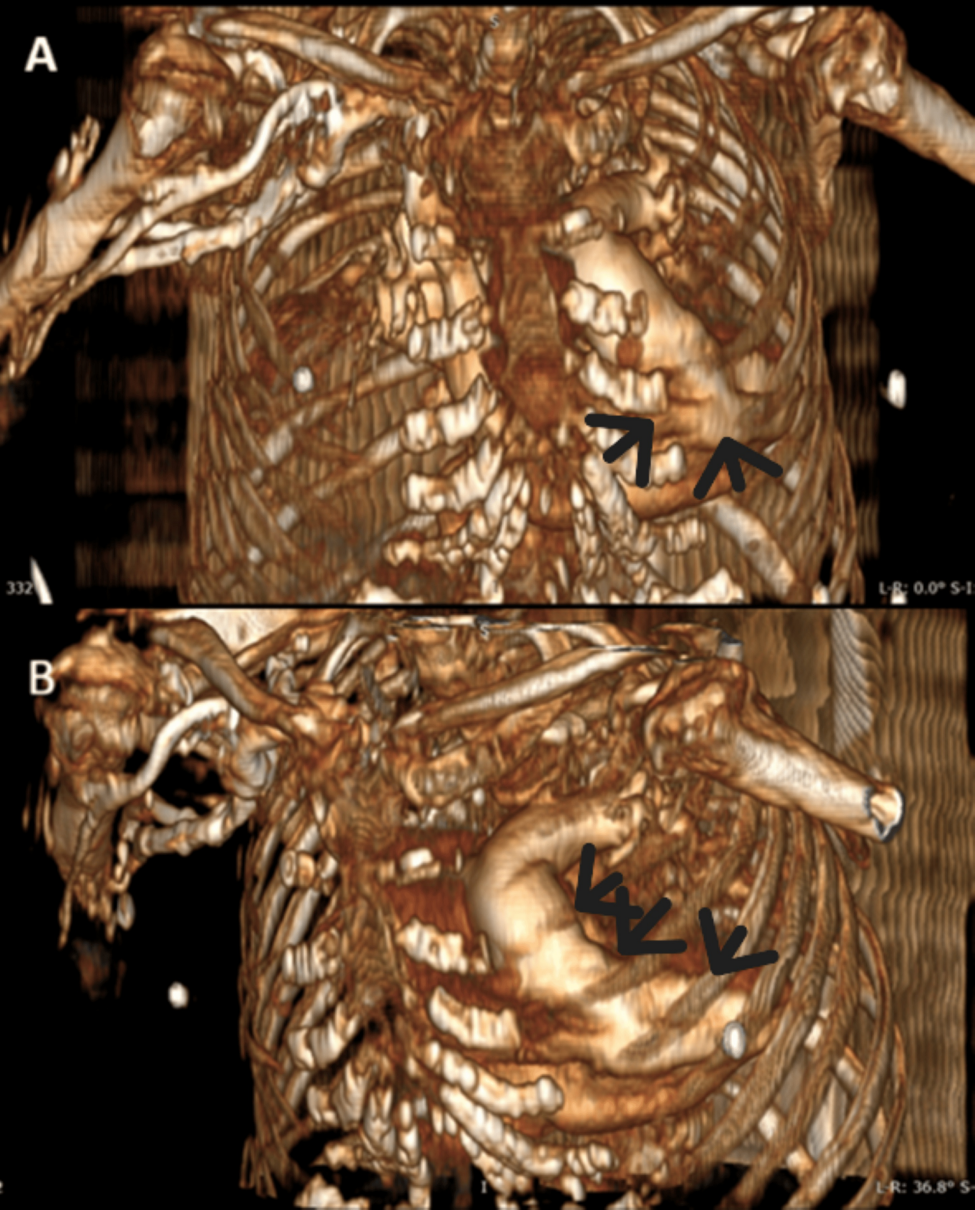
3D CT thoracic reconstruction demonstrating upward and rightward apex (black arrows) orientation, A: frontal view, B: oblique view.

Vector analysis on coronal CT demonstrated a positive vector in lead 1 and augmented vector left (aVL), a negative vector in augmented voltage foot (aVF) (upward rather than downward), and an unusual positive vector in aVR (toward right shoulder), indicating an axis shifted rightward and upward (Figure 3). These findings correlated with the patient’s ECG abnormalities. Three-dimensional reconstruction images confirmed the new cardiac position (Figure 5).

The patient was managed conservatively for her hiatal hernia and discharged with outpatient follow-up. No cardiology-specific intervention was deemed necessary.

## Discussion

Large hiatal hernias, particularly type III, can markedly distort mediastinal anatomy and displace the heart from its normal position. In this patient, the hernia caused an upward and slight rightward displacement of the heart, producing the appearance of elevated apex orientation. This positional alteration accounted for both the atypical imaging findings and the unusual electrocardiographic pattern observed [3-5].

The most notable electrocardiographic feature was a prominent R wave in lead aVR, accompanied by axis deviation. Initially, this raised suspicion for technical issues such as limb lead reversal, conduction abnormalities, or myocardial ischemia [6]. However, the reproducibility of the ECG pattern on repeated tracings, combined with persistently negative cardiac biomarkers, made these possibilities unlikely. CT imaging provided diagnostic clarity by demonstrating that the altered cardiac orientation redirected the main depolarization vector toward the right shoulder, resulting in an upright QRS complex in aVR [7]. Normally, lead aVR records a negative deflection because the cardiac axis points leftward and inferiorly. In this case, the cardiac position with upward and rightward apex orientation shifted the vector upward and rightward, explaining the atypical ECG pattern [6,8].

Reports describing the coexistence of cardiac malposition and a large hiatal hernia are rare. Previous case series have documented that sizable paraoesophageal hernias can compress or displace the heart, occasionally simulating ischemic changes on ECG [9]. Therefore, recognizing the pathologic cardiac anatomy is essential, particularly in elderly patients presenting with nonspecific chest symptoms, abnormal ECGs, or mediastinal widening on imaging [10].

This case underscores the diagnostic value of cross-sectional imaging, particularly CT, in elucidating unexpected ECG findings and preventing unnecessary invasive investigations or interventions. Without imaging correlation, the observed ECG abnormalities might have prompted an unwarranted ischemic workup.

## Conclusions

Cardiac position with an upward and rightward apex orientation is an uncommon finding, particularly in elderly patients. When accompanied by a large hiatal hernia, the resulting mediastinal displacement can complicate both anatomical evaluation and electrocardiographic interpretation. Careful correlation between imaging and ECG findings is essential to ensure accurate diagnosis and to prevent unnecessary or inappropriate management.
